# Methods for siRNA-mediated Reduction of mRNA and Protein Expression in Human Placental Explants, Isolated Primary Cells and Cell Lines

**DOI:** 10.1016/j.placenta.2008.10.003

**Published:** 2009-02

**Authors:** K. Forbes, M. Desforges, R. Garside, J.D. Aplin, M. Westwood

**Affiliations:** Maternal & Fetal Health Research Group, School of Clinical & Laboratory Sciences, University of Manchester, St. Mary's Hospital, Hathersage Road, Manchester M13 0JH, UK

**Keywords:** BeWo, Syncytiotrophoblast cells, First trimester placenta, Transfection, Placental alkaline phosphatase, siRNA

## Abstract

The use of RNA interference (RNAi) to deplete individual proteins from cells or tissue has revolutionised our ability to characterise gene function. The placenta is an attractive target for studies in which the role of specific proteins can be compared with cell culture models and explanted villous tissue where physiological function can be maintained ex vivo.

In this study, we compared a variety of commercially available reagents and approaches to define methods for efficient delivery of siRNA to placental cells. Protocols optimised using fluorescently-labelled siRNA were subsequently tested using siRNA sequences that target placental alkaline phosphatase (PLAP), chosen because of its high abundance in trophoblast. mRNA abundance was assayed using qRT-PCR, and the effect on protein was examined using immunolocalisation.

We report that different protocols are required for BeWo choriocarcinoma cells (nucleofection), primary cytotrophoblast cells (lipid-based transfection) and villous tissue explants (nucleofection). The results provide guidelines for optimal siRNA-mediated knockdown in these three models of the human placenta.

## 1 Introduction

Deletion and mutation have been widely used to examine single gene function in mouse, where more than 100 genes that are required for placental development have been identified [1]. However, to date, only a small fraction of these has been shown to be important for human placental development or function. It is of interest to develop methods capable of effectively blocking specific gene products in the human placenta, both for basic investigations of gene function and future therapeutic approaches to pregnancy pathologies such as abnormal fetal growth. The use of inhibitors can be problematic as availability is limited and many are non-specific and have multiple cellular targets. Targeted reduction of mRNA by RNA interference (RNAi) offers an attractive alternative approach to reducing the abundance of the corresponding protein in order to examine function in living cells.

RNA interference by short interfering strands (siRNA) is a normal post-translational regulatory mechanism and a vital part of the innate immune response, acting as a defense mechanism against viruses by inhibiting gene expression at the stage of translation [2]. RNAi can be artificially induced in mammalian cells by introducing synthetic siRNA (21–23 base pairs in length) or plasmid or viral vectors expressing short hairpin RNAs (shRNAs) [3]. Many different methodologies, including lipid-based transfection, retroviruses and electroporation have been used for *in vitro* delivery of siRNA into cells and tissue [4], however the efficiency of delivery varies amongst cell types and optimisation of conditions can be expensive and time-consuming.

In this study we have used non-targeted fluorescently-labelled siRNA sequences as an initial approach to assay efficiency of methods for transfecting BeWo choriocarcinoma cells, isolated primary cytotrophoblast cells and placental villous explants. We have shown that in each system, the method identified as most efficient produces significant mRNA and protein knockdown using as an exemplar siRNA directed against human placental alkaline phosphatase (PLAP).

## 2 Methods

### 2.1 Determining transfection efficiency

Fluorescently-labelled non-targeting siRNA (siGLO red; 50–100 nM; Dharmacon, UK) was used to assess the efficiency of a variety of methods for introducing siRNA into cells and tissue. Following transfection, samples were incubated for 24–48 h and then cells were fixed in 4% paraformaldehyde for 30 min and tissue embedded in OCT, snap frozen and sectioned (10 μm). All samples were mounted with Vectashield (Vector Laboratories, UK) containing a nuclear counterstain (DAPI) and visualised with a Zeiss AxioObserver inverted microscope (Carl Zeiss Inc, Europe). Transfection was deemed successful when more than 70% of cells contained intracellular fluorescence.

### 2.2 siRNA sequences

In order to demonstrate that the procedure resulting in the most efficient delivery of non-targeting siRNA sequences also allowed the depletion of specific mRNA, cells and tissue were transfected with 100 nM of an siRNA sequence (5′-AACGGTCCAGGCTATGTGCTC-3′; Dharmacon, UK) designed to target specifically mRNA encoding the highly abundant placental protein, placental alkaline phosphatase (PLAP; (GenBank) accession number NM_001632.3) [5]. The sequence was verified by Basic Local Alignment Search Tool (BLAST). The effect of targeted (PLAP) siRNA sequences was compared with 100 nM non-targeting Silencer negative control siRNA (Ambion, USA) and AllStars negative control siRNA (Qiagen, UK).

### 2.3 Analysis of PLAP mRNA expression

Total RNA was extracted from placental tissue and from cells using an Absolutely RNA miniprep or microprep kit (Stratagene, USA) respectively, quantified using a Quant-iT Ribogreen kit (Molecular Probes) and 100 ng of total RNA from each sample was reverse transcribed using AffinityScript cDNA synthesis kit (Stratagene, USA). PLAP and β actin mRNA expression were quantified by QPCR using a Stratagene MX3000P real time PCR machine and Stratagene Brilliant SYBR Green I QPCR mastermix, with 5-carboxy-*x*-rhodamine as a passive reference dye. PLAP was amplified using 300 nM primers (Invitrogen UK) designed by Sequence Manipulation Suite Software (http://bioinformatics.org/sms2/pcr_primer_stats.html) and confirmed as specific by BLAST analysis: (forward: 5′ GCTCATACTCCATGCCCA 3′, reverse: 5′ AGACACCCCCATCCCATC 3′). Primers for β actin mRNA (200 nM; MWG Biotech (UK)) were: forward: 5′-AGCCACCCCACTTCTCTCTAA-3′, reverse: 5′-ACACGAAAGCAATGCTATCACCT-3′ [6]. PLAP and β actin mRNA were quantified against standard curves generated from total human placental RNA and human reference total RNA (Stratagene, La Jolla, USA) respectively. Data were analysed by using the Kruskal–Wallis test and are presented as median and range mRNA expression relative to the control (untransfected) sample for the corresponding experiment.

### 2.4 Analysis of PLAP protein expression

Following treatment with siRNA, placental tissue was embedded in OCT, snap frozen and sectioned (10 μM). BeWo cells and primary cytotrophoblast cells were fixed in ice-cold methanol. Expression of PLAP protein was assessed by incubating tissue or cells with mouse monoclonal anti-PLAP antibody (1:500; Sigma clone 8B6) for 1 h and then FITC-labelled rabbit anti-mouse IgG (46 μg/ml; DakoCytomation) for 1 h before counterstaining with propidium iodide (PI) and capturing images using a Zeiss AxioObserver Inverted Microscope.

### 2.5 Establishing optimal siRNA delivery methods

#### 2.5.1 BeWo cells

BeWo choriocarcinoma cells were cultured in Dulbecco's modified Eagle's medium (DMEM)/Ham's F12 (F12) (1:1, v/v) containing 10% fetal calf serum and antibiotics, as previously described [7]. In order to test lipid-based transfection reagents, BeWo cells were plated on coverslips in 24-well plates and, when approximately 40–50% confluent, transfected with either Lipofectamine 2000, Oligofectamine (Invitrogen, UK), or Fugene HD (Roche Diagnostics, UK) transfection reagents in accordance with the manufacturer's guidelines. Cells destined for transfection using an electroporation-based system - nucleofection, were grown in T75 flasks until 70–80% confluent, then trypsinised and counted. 1 × 10^6^ cells/condition were resuspended in cell line solution L or cell line solution V and transfected following the manufacturers' instructions using programs A-020, T-020, X-001 and X-005 (Amaxa Biosystems, Germany). Following transfection, cells were plated at approximately 7 × 10^4^/well for 24-well plates and approximately 3 × 10^5^/well for 6 well plates.

#### 2.5.2 Primary cytotrophoblast cells

Term placentae (38–40 weeks gestation) were collected with written informed consent and in accordance with Local Ethics Committee approval following Caesarean section or vaginal delivery from uncomplicated singleton pregnancies. Cytotrophoblast cells were isolated and maintained in primary culture for up to 90 h as described previously [8,9].

In order to test lipid-based transfection reagents, cytotrophoblast cells were plated onto 12 well plates at approximately 2 × 10^6^/well and after 18 h in culture, transfected using either Lipofectamine LTX (Invitrogen, UK) or DharmaFECT2 (Dharmacon, UK) reagents in accordance with the manufacturers' guidelines.

Cells intended for transfection by nucleofection were centrifuged at 1000 g for 5 min immediately after isolation and then resuspended in the nucleofection buffer (100 μl/2 × 10^6^ cells) provided in the basic primary mammalian epithelial cell nucleofector kit (Amaxa Biosystems, Germany). 2 × 10^6^ cells/condition were transfected using programme Nucleofector programmes S-005, T-020, T-023 or U-017 and then plated onto 12 well plates at 1–2 × 10^6^ cells/well in 1.5 ml culture medium (DMEM/Ham's F12 (1:1, v/v) containing 10% fetal calf serum and antibiotics).

#### 2.5.3 1st trimester human placenta explants

First trimester (8–12 weeks gestation) placentae were obtained with written informed consent and in accordance with Local Ethics Committee approval, following elective surgical or medical termination of pregnancy. Fresh villous tissue was dissected under sterile conditions in serum-free DMEM/F12 and small (approximately 5 mm) pieces were transfected and then transferred to 1% agarose-coated 24-well tissue culture plates for up to 4 days in 20% O_2_ at 37 °C. Our previous studies suggest that tissue maintained under these conditions displays similar morphology and functional characteristics to first trimester tissue cultured at lower oxygen concentrations [10], however researchers wishing to perform experiments at ≤20% oxygen should consider the possibility that oxygen tension may impact on the efficiency of siRNA-mediated knockdown of mRNA/protein.

Lipofectamine 2000, Oligofectamine, Fugene HD, and DharmaFECT2 were tested, each in accordance with the manufacturer's instructions, as lipid-based methods for introducing siRNA sequences into placental explants. In order to carry out transfection by nucleofection, 2–3 pieces of placental tissue were placed in 100 μl basic primary epithelial cell nucleofector buffer containing siRNA or vehicle. Using a wide-ended 1.5 ml Pasteur pipette, tissue and buffer were transferred to a Nucleofector cuvette and then exposed to programs X005, X001, U006, U007 and U017 before removal using pipettes supplied with the Nucleofector kit.

## 3 Results

### 3.1 BeWo cells

**Fig. 1:**
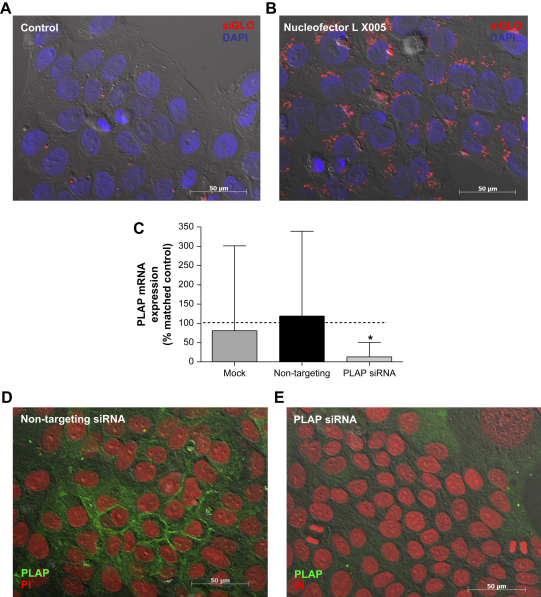
Delivery of siRNA to BeWo cells. 24 h after transfection cells were fixed and stained with DAPI (blue) **(A)**; optimal delivery of fluorescently-labelled siRNA (red) was achieved using an Amaxa Nucleofector with cell line solution L and program X005 **(B)**. **(C)** Cells exposed to transfection conditions in the absence of siRNA (mock) or cells transfected with non-targeting (100 nM) or PLAP-specific siRNA (100 nM) were cultured for 72 h and then analysed for PLAP mRNA expression (median and range, *n* = 7). Raw data were analysed by using the Kruskal–Wallis test (*p < 0.05 versus control cells) and are presented as median and range mRNA expression relative to the control (untransfected) sample for the corresponding experiment. Expression of PLAP protein (green) was analysed 72 h after transfection with non-targeting **(D)** or PLAP-specific siRNA **(E)**; nuclei are stained with PI (red).

Relatively low percentages of fluorescent cells were observed after BeWo cells were transfected using Oligofectamine (approx 40%) or Fugene HD (<40%). Approximately 50% of cells were transfected when Lipofectamine 2000 was used to deliver siRNA, however a high degree of cell death was observed (Table 1). Optimal transfection (>90% fluorescent-positive cells in conjunction with good cell viability) was achieved with the Amaxa Nucleofector using cell line solution L and program X005 (Fig. 1A and B; Table 1).

In order to confirm that this methodology gave knockdown of target proteins, BeWo cells were transfected with non-targeting or PLAP-specific siRNA. PLAP mRNA expression was reduced by 75% after 72 h (*p* < 0.05; Fig. 1C; Table 1) and immunohistochemical analysis demonstrated a corresponding reduction in PLAP protein expression in both cytoplasmic and cell surface-associated pools (Fig. 1D and E; Table 1). The level of β actin mRNA in BeWo cells was not affected by transfection with PLAP-specific siRNA (data not shown).

### 3.2 Primary term cytotrophoblast cells

**Fig. 2:**
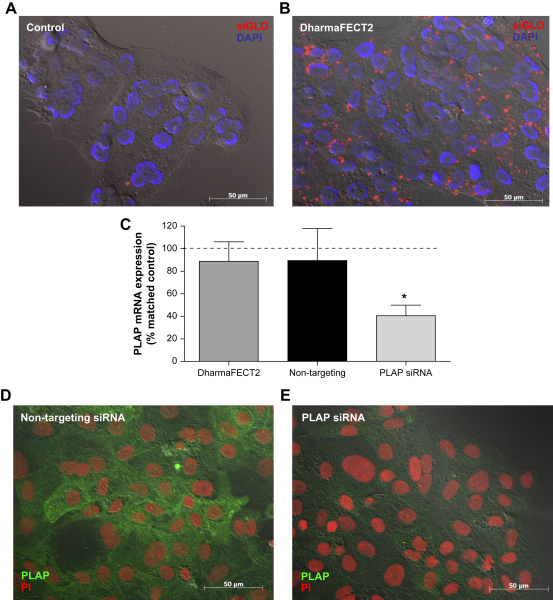
Delivery of siRNA to primary term cytotrophoblast cells. 18 h after isolation, primary cytotrophoblast cells were transfected with fluorescent-labelled siRNA (red) and then 48 h later, cells were fixed and stained with DAPI (blue). Images show distribution of siRNA in control cells **(A)** or cells transfected with DharmaFECT2 **(B)**. **(C)** Mock-transfected (DharmaFECT2) cells or cells transfected with non-targeting (100 nM) or PLAP-specific siRNA (100 nM) were cultured for 48 h and then analysed for PLAP mRNA expression (*n* = 5). Raw data were analysed by using the Kruskal–Wallis test (*p < 0.05 versus control cells) and are presented as median and range mRNA expression relative to the control (untransfected) sample for the corresponding experiment. Expression of PLAP protein (green) was analysed 48 h after transfection with non-targeting **(D)** or PLAP-specific siRNA **(E)**; nuclei are stained with PI (red).

The nucleofector was unsuitable for transfecting primary cytotrophoblast cells since this method resulted in high levels of cell death and only a few of the surviving cells were fluorescently-labelled (Table 1). High levels of intracellular fluorescence (approximately 80% positive cells) were observed following transfection with Lipofectamine LTX, however there was some evidence (nuclear fragmentation) of increased apoptosis (Table 1). Approximately 95% of cells were fluorescence-positive when DharmaFECT2 was used to deliver siRNA (Fig. 2A and B; Table 1). Transfection with DharmaFECT2 was selected as the optimal method for delivering siRNA sequences to cytotrophoblast cells. PLAP mRNA expression was not affected by transfection with non-targeting siRNA but mRNA levels were reduced by approximately 60% 48 h after delivery of PLAP-specific siRNA (*p* < 0.05; Fig. 2C; Table 1). Similarly, delivery of PLAP siRNA with DharmaFECT2 resulted in reduced PLAP protein expression as demonstrated by immunocytochemistry (Fig. 2D and E; Table 1). Transfection of cytotrophoblast cells with PLAP siRNA did not affect expression of β actin mRNA (data not shown).

### 3.3 First trimester placental explants

**Fig. 3:**
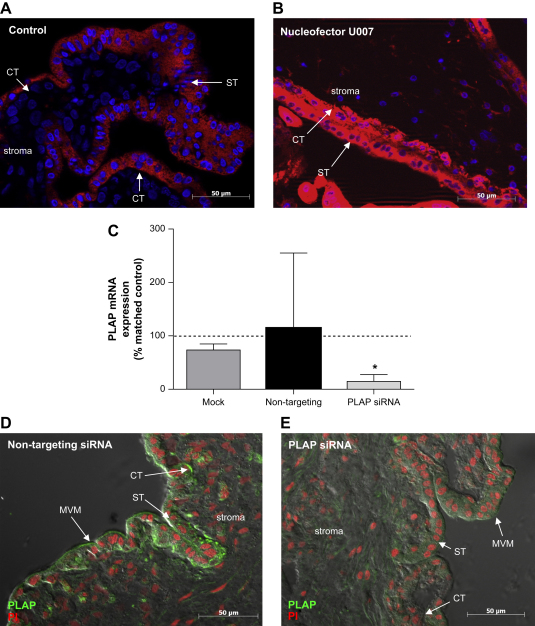
Delivery of siRNA to first trimester placental explants. 24 h after transfection tissue was fixed and stained with DAPI (blue). Images show distribution of fluorescently-labelled siRNA (red) in (A) control tissue (no transfection reagents) and (B) following transfection with the Amaxa Nucleofector (basic mammalian epithelial cell solution; program U007). Arrows indicate cytotrophoblast cells (CT), syncytium (ST) and stroma. (C) Tissue exposed to transfection conditions in the absence of siRNA (mock) or tissue transfected with non-targeting (100 nM) or PLAP-specific siRNA (100 nM) was cultured for 72 h and then analysed for PLAP mRNA expression (*n* = 4). Raw data were analysed by using the Kruskal–Wallis test (*p < 0.05 versus control cells) and are presented as median and range mRNA expression relative to the control (untransfected) sample for the corresponding experiment. Expression of PLAP protein (green) was analysed 48 h after transfection with non-targeting (D) or PLAP-specific siRNA (E); nuclei are stained with PI (red).

In control tissue (no transfection reagents) siRNA could gain access to the syncytial layer of the placenta. However, neither the underlying cytotrophoblast cells nor villous stromal cells were positive for fluorescently-labelled siRNA (Fig. 3A; Table 1). When lipid-based transfection reagents were utilised, the transfected tissue resembled control tissue, with siRNA present only in the syncytium. However, when tissue was transfected using the nucleofector, high levels of siRNA were observed in both cytotrophoblast cells and in the villous stroma (basic mammalian epithelial cell solution, program U007; Fig. 3B; Table 1). Preliminary experiments investigated if nucleofection could also be used to transfect cytotrophoblast following removal of syncytium by limited trypsinisation. However, cell viability was severely impaired following sequential manipulation and the approach was discontinued. As transfection of intact tissue by nucleofector resulted in delivery of siRNA sequences to cytotrophoblast cells and stroma in addition to syncytium, this method was chosen for further evaluation.

PLAP mRNA expression was significantly reduced following transfection with PLAP siRNA (85%, *p* < 0.05; Fig. 3C; Table 1). Neither mock nor non-targeting siRNA had any effect on PLAP mRNA levels. Immunofluorescence revealed that PLAP protein expression (in microvillous membrane and cytotrophoblast cells, as indicated by arrows) was markedly reduced by PLAP siRNA (Fig. 3D and E; Table 1). The expression of β actin mRNA was not affected by nucleofector-mediated transfection of placental explants (data not shown).

## 4 Discussion

Different techniques for introducing siRNA oligonucleotides into human placental cells and tissue offer varying degrees of success in terms of transfection efficiency. We have optimised delivery of siRNA sequences into placental cell lines, isolated cells and tissue, and demonstrated efficient knockdown at both mRNA and protein level of the highly abundant protein, placental alkaline phosphatase.

Most published studies reporting siRNA or antisense oligonucleotide-mediated knockdown in the human placenta have focussed on cell lines. Though cell lines offer advantages including convenience and ease of transfection, reported transfection efficiencies vary, and for studies of normal placental function it is preferable to study primary trophoblast cells and tissue explants. We are unaware of previous reports of siRNA delivery into primary cytotrophoblast cells and there are limited available data in placental tissue.

Calcium phosphate-assisted transfection is a relatively easy and inexpensive method for introducing RNA or DNA but is reported to show very low efficiency in placental cells [11]. Limited delivery was achieved with either poly-L-ornithine or DEAE-dextran (25% and 6.25% respectively [11]). More recently siRNA or plasmid DNA has been introduced into placental cell lines such as JAR, JEG or BeWo using Lipofectamine, however many of these studies do not report transfection efficiency [12,13], whilst others report efficiencies varying in the range 50–80% using Lipofectamine [14,15]. In our hands Lipofectamine can be used to introduce siRNA into BeWo cells however, the transfection efficiency is low and subsequent analysis revealed little effect on protein level (data not shown).

Numerous reagents have been used to deliver siRNA into primary cells, which are generally considered to be hard to transfect. Zhang et al. [16] have reported successful use of Lipofectamine 2000 in human placental fibroblasts. However, their procedure involves a trypsinisation step prior to siRNA delivery which is unsuitable for primary human cytotrophoblast cells, as once isolated and cultured, these cells are non-proliferative and do not survive further rounds of trypsinisation. We have found that DharmaFECT2 transfection reagent applied to 18 h primary human cytotrophoblast cells in culture is an effective method for introducing siRNA into primary human cytotrophoblast.

In the absence of delivery agents, siRNA spontaneously enters the syncytium of first trimester villous tissue, but cannot access underlying cytotrophoblast cells or the villous stroma. This does enable knockdown of syncytial proteins, as recently demonstrated by the reduction of p53 and mdm2 in term placenta [15]. In addition to syncytium, PLAP is expressed in cytotrophoblast cells and to some extent in stromal cells; here we found only a small reduction in protein expression without active delivery of siRNA. The nucleofector-based method we describe delivers siRNA to placental explants to produce knockdown in cell populations beneath the syncytium.

In this study we have used PLAP to demonstrate that an abundant placental protein can be depleted using each of the optimal delivery methods described. Other gene products may behave differently depending on siRNA design and half-life of target mRNA, hence specific optimisation is recommended before undertaking functional experiments, including target sequences, siRNA concentrations and exposure times [17].

In summary, this study highlights the importance of validating delivery methodologies to obtain optimal conditions when using siRNA. We have demonstrated that effective delivery to primary cytotrophoblast cells is by transfection with DharmaFECT, whilst the optimal conditions for introducing siRNA into BeWo cells and placental explant tissue (beyond the syncytiotrophoblast) is by electroporation using a nucleofector. As new technologies emerge, improved siRNA delivery may be expected. At present however, the techniques described are suitable for studying the role of individual genes and their translation products *in vitro* and will aid in the analysis of placental development and function. Future possibilities include the introduction of RNAi into the placenta as a therapeutic strategy for pregnancy complications and diseases with fetal onset.

## Figures and Tables

**Table 1:** Summary of cell/tissue characteristics following introduction of siGLO/siRNA sequences via a number of different methods.

Cell type	Analysis	No Reagent	Lipid-Based transfection	Nucleofection	
				*Cell Line Solution L*	*Cell Line Solution V*
BeWo	Transfection efficiency (% cells containing siGLO)	0%	Lipofectamine 2000: 50% Oligofectamine: 40% Fugene HD: <40%	Prog A020: 60% Prog T020: 50% Prog X001: >70% Prog X005: >90%	Prog A020: 60% Prog T020: 50% Prog X001: >70% Prog X005: >70%

	Cell Survival (%)	85%	Lipofectamine 2000: 40% Oligofectamine: 70% Fugene HD: 70%	Prog A020: 50% Prog T020: 50% Prog X001: 60% Prog X005: >70%	Prog A020: 50% Prog T020: 50% Prog X001: 60% Prog X005: 60%

	PLAP mRNA (%)	N/A	N/A	Prog X005: 75%	N/A
	PLAP protein (%)	N/A	N/A	Prog X005: >70%	N/A

				*Basic Mammalian Epithelial Cell Solution*	
Primary trophoblast	Transfection efficiency (% cells containing siGLO)	50%	Lipofectamine LTX: 80% DharmaFECT2: 95%	Prog S005: Most of surviving cells Prog T020: Most of surviving cells Prog T023: Most of surviving cells Prog U017: Most of surviving cells	
	Cell Survival (%)	85%	Lipofectamine LTX: 40% DharmaFECT2: 85%	Prog S005: <30% Prog T020: <30% Prog T023: <30% Prog U017: <30%	
	PLAP mRNA (%)	N/A	DharmaFECT2: 60%	N/A	
	PLAP protein (%)	N/A	>70%	N/A	

1st Trimester placental tissue	Transfection efficiency (% cells containing siGLO)	100% syncytium 0% cytotrophoblast 0% stromal cells	Lipofectamine 2000, Oligofectamine, Fugene HD all gave similar transfection efficiencies:100% syncytium; 0% cytotrophoblast; 0% stromal cells	Prog X005: 100% syncytium; 70% cytotrophoblast; 30% stromal cells Prog X001: 100% syncytium; 50% cytotrophoblast; <10% stromal cells Prog U017: 100% syncytium; 50% cytotrophoblast; 10% stromal cells Prog U007: 100% syncytium; 80% cytotrophoblast; 40% stromal cells	
	Cell Survival (%)	>90%	N/A	Prog U007: >70%	
	PLAP mRNA (%)	N/A	N/A	Prog U007: 85%	
	PLAP protein (%)	<20%	N/A	Prog U007: >60%	
